# Supplementary material for: Phenotypic memory in Bacillus subtilis links dormancy entry and exit by a spore quantity-quality tradeoff
Source: Nat Commun. 2018 Jan 4;9:69. doi: 10.1038/s41467-017-02477-1 (PMC5754360; doi:10.1038/s41467-017-02477-1)
Supplement: Supplementary file 1 — Supplementary Information [file 41467_2017_2477_MOESM1_ESM.pdf]

Supplementary Figures

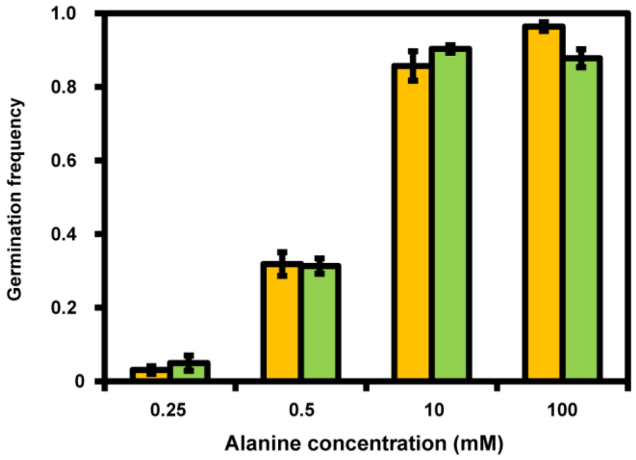

Supplementary Figure 1. *Early* and *late* spores achieve comparable germination frequencies. Germination frequency of *early* (yellow) and *late* spores (green) as a function of the L-alanine concentration that was used for stimulating spores after sporulation on agarose pads. See Methods for details. Results denote the final germination frequency after 2 hours upon which no further germination events were observed in the microcolonies. Strain: BIB1019. Data: mean ± SEM, $n_c = \geq 4$ ($n_s = \geq 200$). Unpaired t-test: the differences between *early* and *late* spores are not significant ($P > 0.05$).

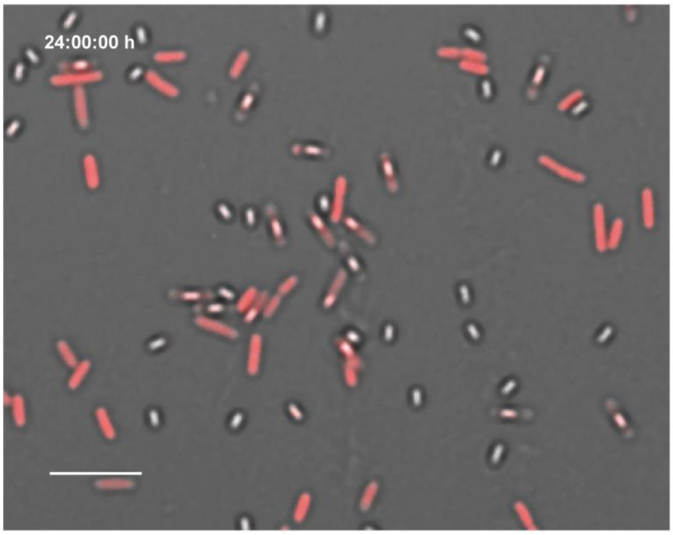

Supplementary Figure 2. Micrograph of P_{rapA}-*mCherry* (BIB1126) cells from a culture that was cultivated for 24 h in liquid SM. Spores that have been released from the sporangia do not show any fluorescence (*early* spores). Spores contained within mother cells show some fluorescence, cells that delay sporulation for even longer are strongly fluorescent. Scale bar: 5 µm.

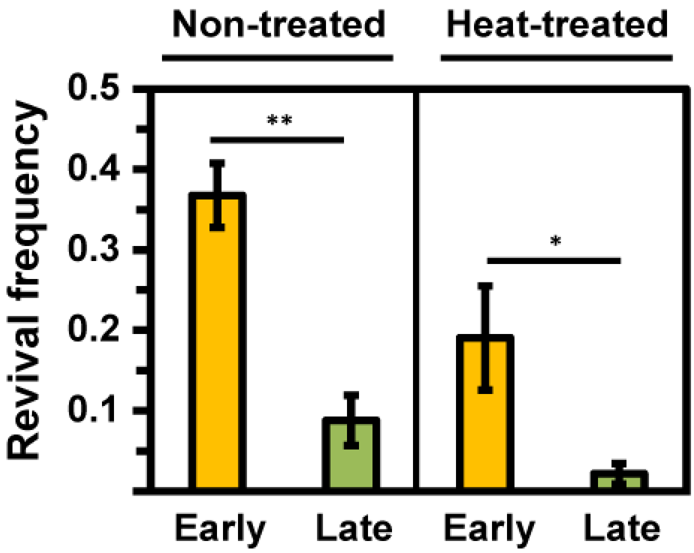

Supplementary Figure 3. Effect of heat-treatment on differential spore revival. Revival frequencies of *early* and *late* spores (BIB1126) which were heat-treated prior to induction with L-alanine on a SM pad. Data: mean ± SEM, n = 4 movies (n_s = ≥ 700), unpaired t-test: * $P \leq 0.05$, ** $P \leq 0.01$.

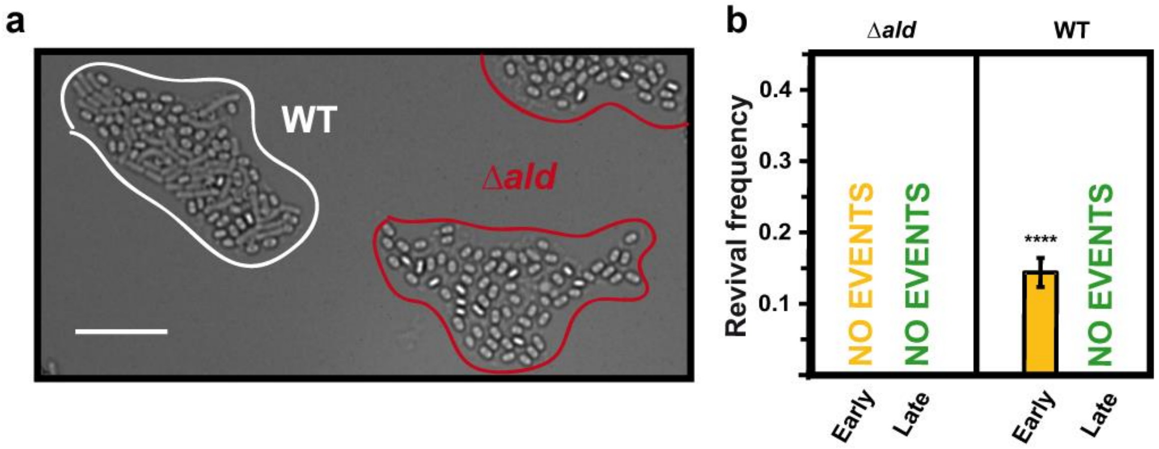

Supplementary Figure 4. Effect of an *ald* gene knock-out. (a) Bright-field image of a colony of an *ald* knock-out (BIB1416) that was co-cultured with a WT strain (BIB224). The *ald* strain carries the P_{trpE*}-*mCherry* promoter fusion to distinguish it from the WT. A representative image of spore colonies at t=10 h *after* stimulation with L-alanine is shown. Several *early* WT spores have grown out successfully, while most *ald* mutant spores have germinated but none have grown out. See also Supplementary Movie 4. Scale bar: 10 µm. **(b)** Corresponding revival frequencies. Strains: BIB1416, BIB224. Data: mean ± SEM, $n_c = \geq 8$ ($n_s = \geq 500$), unpaired t-test: **** $P \leq 0.0001$.

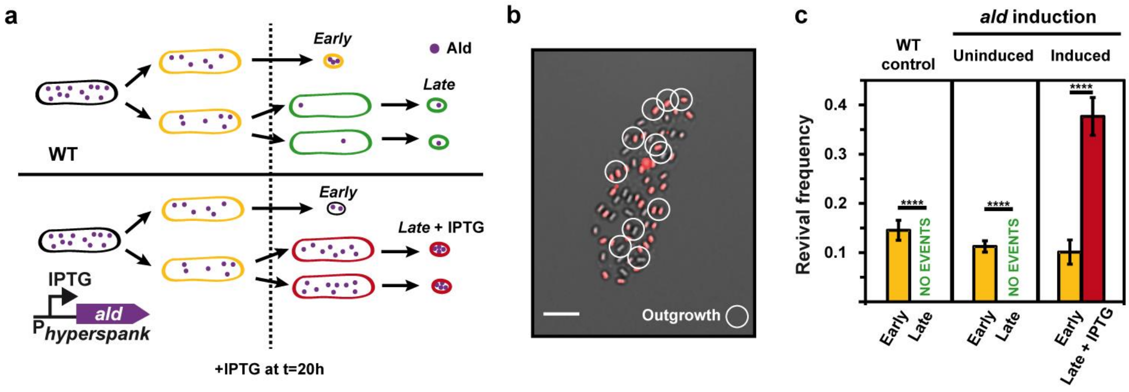

Supplementary Figure 5. Effect of Ald-induction in the progenitor cells of *late* spores. (a) Schematics illustrating the formation of *late* spores carrying low levels of Ald in the WT (top) and higher levels of Ald (bottom) formed by inducing *ald* expression from a σ^A -dependent promoter specifically in the progenitors of *late* spores. **(b)** Micrograph of a spore microcolony showing Ald-induced *late* spores in red. The bright-field image (gray) was overlaid with a fluorescence image of the P_{rapA}-*mCherry* reporter. Spores that grew out in response to L-alanine are circled. See also Supplementary Movie 5. Scale bar: 5 µm. **(c)** Corresponding revival frequencies of *early* and *late* spores in response to L-alanine. Results from left to right show the WT, the uninduced control, and the induced strain as shown in (a) and (b). Strains: BIB1300 and BIB224 (WT), Data: mean ± SEM, $n_c = \geq 11$ ($n_s = \geq 800$), unpaired t-test: **** $P \leq 0.0001$.

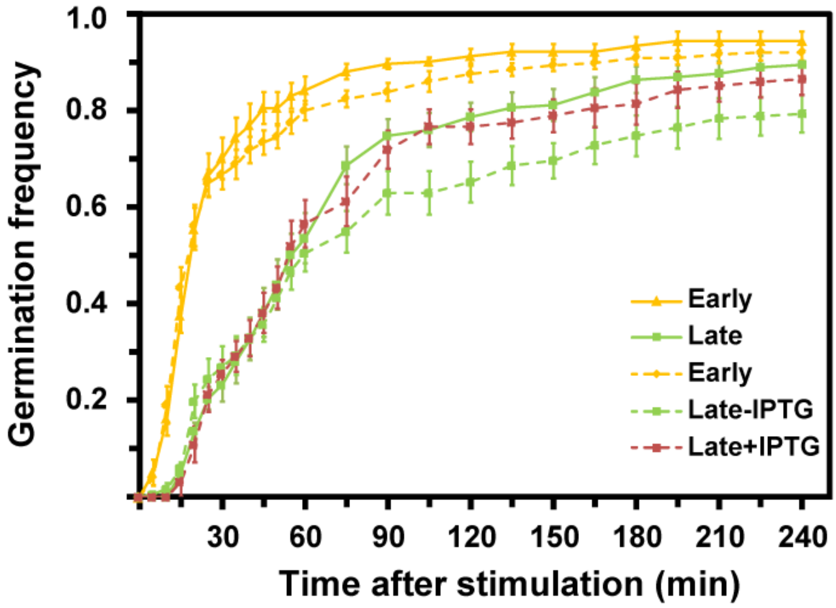

Supplementary Figure 6. Ald does not affect the germination response to L-alanine. Germination frequency as a function of time after stimulating spore microcolonies with L-alanine at time t = 0. During the nutrient down-shift Ald expression was induced at t = 30 h in the progenitor cells of (very) *late* spores (red). Their germination dynamics were comparable to WT *late* spores (green line) or *late* spores that had formed before the induction of Ald expression, i.e. between t = 20 h and t = 30 h (green dashed line). The germination dynamics of *early* spores (yellow) are included as a reference in each case. Strain: BIB1300 and BIB224 (WT), Data: mean ± SEM, $n_c = \geq 8$ ($n_s = \geq 350$).

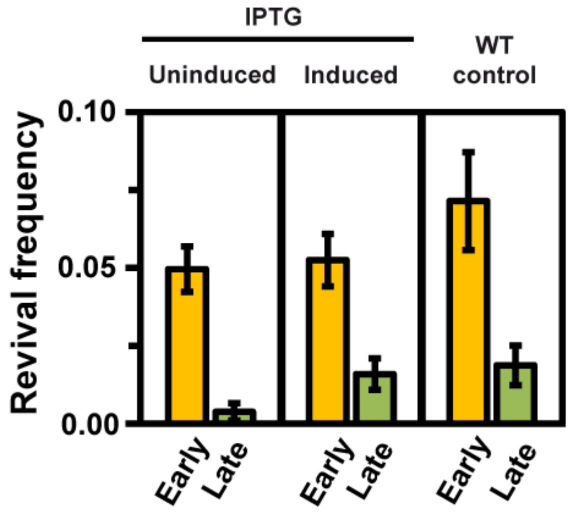

Supplementary Figure 7. Ald does not affect the spore revival frequency in response to stimulation with AGFK. Revival frequency (after 8 h) in response to stimulation with AGFK at $t = 0$ h. During sporulation BIB1300 was induced with IPTG at $t = 20$ h and compared to the uninduced control. The data for the WT strain is included for reference. Strains: BIB1300, BIB224 (WT). Data: mean ± SEM, $n_c = \geq 12$ ($n_s = \geq 900$). Unpaired t-test: The difference between induced and uninduced spores is not significant ($P > 0.05$).

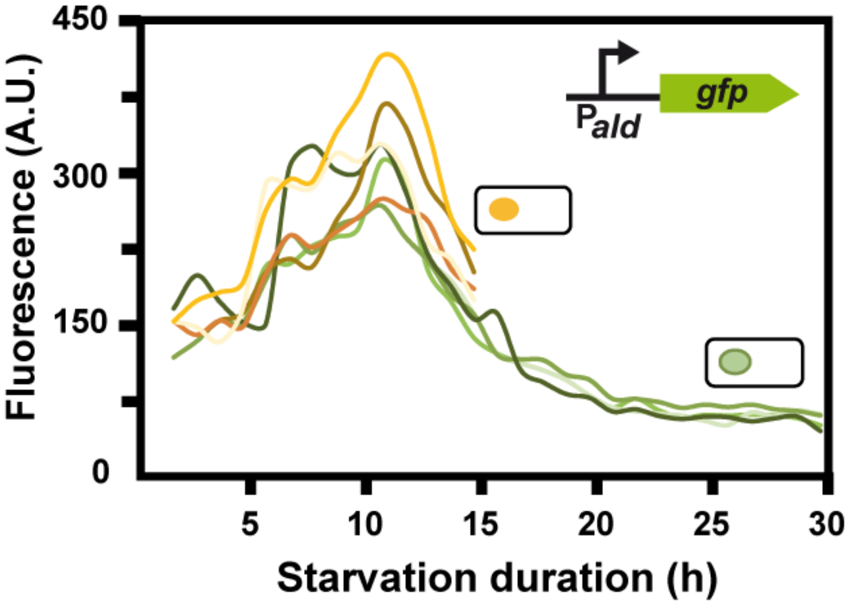

Supplementary Figure 8. Cells down-regulate the *ald* promoter during starvation. Fluorescence trajectories for single cells carrying a P_{ald}-*gfp* reporter (BIB1213). Yellowish (greenish) denote the trajectories for cells that give rise to *early* (*late*) spores. The trajectories were terminated when a pre-spore became visible.

Supplementary Tables

Supplementary Table 1. Vectors used in this work

Accession Number	Vector	Genotype	Source
**EIB380**	pGFP_Star	‘*amyE cat* T*gyrA* LICS promoterless *gfpmut3 amyE*’ *bla* ColE1 origin	Ref. 1
**EIB422**	pRFP_Star	‘*amyE cat* T*gyrA* LICS promoterless *mcherry amyE*’ *bla* ColE1 origin	This work
**EIB303**	pDR111	‘*amyE spec*^{R} T*rrnB* T$_0$λ *Phyperspank-MCS lacI amyE*‘ *bla* ColE1 origin	David Rudner, Harvard Medical School
	pDR110	‘*amyE spec*^{R} T*rrnB* T$_0$λ *Pspank-MCS lacI amyE*‘ *bla* ColE1 origin	David Rudner, Harvard Medical School
**EIB7**	pSac-KAN	‘*sacA kan*^{R} *MCS sacA*’ *bla* puc18 origin	Ref. 2
**EIB25**	pDG1514	‘*MCS par tet*^{R} *lacZ*’ *bla* pMB1 origin	Ref. 3

Supplementary Table 2. Plasmids used in this work

Accession Number	Plasmid	Genotype	Source
**EIB432**	pRFP_Star_P$_{rapAII}$	‘*amyE cat*^{R} T*gyrA* P$_{rapAII}$-*mcherry amyE*’ *bla* ColE1 origin	This work
**EIB404**	pGFP_Star_P$_{spoIIE}$	‘*amyE cat*^{R} T*gyrA* P$_{spoIIE}$-*gfpmut3 amyE*’ *bla* ColE1 origin	This work
**EIB450**	pGFP_Star_P$_{ald}$	‘*amyE cat*^{R} T*gyrA* LICS P$_{ald}$-*gfpmut3 amyE*’ *bla* ColE1 origin	This work
**EIB499**	pGFP_Star_P$_{ald}$-*ald-mCherry*	‘*amyE cat*^{R} P$_{ald}$-*ald-mCherry* T*gyrA* P$_{ald}$-*gfpmut3 amyE*’ *bla* ColE1 origin	This work
**EIB452**	pDR111_*ald*	‘*amyE spec*^{R} P$_{hyperspank}$-*ald lacI amyE*’ *bla* ColE1 origin	This work
**EIB297**	pDR111_*rapA*	‘*amyE spec*^{R} P$_{hyperspank}$-*rapA lacI amyE*’ *bla* ColE1 origin	This work
**EIB419**	pDR110_*kinA* / 04F14	‘*amyE spec*^{R} P$_{spank}$-*kinA lacI amyE*‘ *bla*ColE1 origin	Ref. 4
**EIB480**	pSac-KAN_ P$_{hyperspank}$-*ald*	‘*sacA* P$_{hyperspank}$-*ald lacI kan*^{R} *sacA*’ *bla* ColE1 origin f1(+) origin	This work
**EIB503**	pSac-KAN_P$_{ald}$-*ald-mCherry*	‘*sacA* P$_{ald}$-*ald-mCherry lacI kan*^{R} *sacA*’ *bla* ColE1 origin f1(+) origin	This work

Supplementary Table 3. Strains used in this work

Strains	Name	Genotype	Source
E. coli			
	DH5α	*F^- φ80lacZΔM15 Δ(lacZYA-argF)U169 recA1 endA1 hsdR17(r_k^-, m_k^+) phoA supE44 thi-1 gyrA96 relA1 λ^-*	Invitrogen
B. subtilis			
**BIB224**	168 1A700	*trpC2*	BGSC
**BIB182**	168 P_{trpE*}-*mcherry* P_{spo0F}-*yfp* / JL028	*amyE*::[P_{spo0F}-*yfp* cat^R] *ppsB*::[P_{trpE*}-*mcherry* ery^R]	Ref. 5
**BIB444**	168 P_{trpE*}-*mcherry*	*trpC2 ppsB*::[P_{trpE*}-*mcherry* ery^R]	See methods
**BIB1019**	168 P_{spoIIE}-*gfp* P*trpE-mcherry*	*trpC2* *amyE*::[T*gyrA* P_{spoIIE}-*gfpmut3* cat^R] *ppsB*::[P_{trpE*}-*mcherry* ery^R]	BIB444 transformed with EIB404
**BIB1126**	168 P_{rapAII}-*mcherry*	*trpC2* *amyE*::[T*gyrA* P_{rapAII}-*mcherry* cat^R]	BIB224 transformed with EIB432
**BIB1416**	168 *ald*::tet^R P_{trpE*}-*mcherry*	*trpC2 ald*::tet^R *ppsB*::[P_{trpE*}-*mcherry* ery^R]	See methods
**BIB1213**	168 P_{ald}-*gfp*	*trpC2 amyE*::[T*gyrA* P_{ald}-*gfpmut3 lacI* cat^R]	BIB224 transformed with EIB450
**BIB1300**	168 P_{rapAII}-*mcherry* $P_{hyperspank}$-*ald*	*trpC2* *amyE*::[T*gyrA* P_{rapAII}-*mcherry* cat^R] *sacA*::[$P_{hyperspank}$-*ald lacI* kan^R]	BIB1126 transformed with EIB480
**BIB1423**	168 P_{ald}-*ald-mcherry* P_{ald}-*gfp*	*trpC2* *amyE*::[P_{ald}-*ald-mcherry* cat^R T*gyrA* P_{ald}-*gfpmut3*]	BIB224 transformed with EIB499
**BIB1332**	168 P_{spank}-*kinA*	*trpC2 amyE*::[P_{spank}-*kinA lacI* $spec^R$]	BIB224 transformed with EIB419
**BIB1330**	168 $P_{hyperspank}$-*rapA*	*trpC2 amyE*::[$P_{hyperspank}$-*rapA lacI* $spec^R$]	BIB224 transformed with EIB297
**BIB1440**	168 P_{spank}-*kinA* P_{ald}-*ald-mcherry*	*trpC2* *amyE*::[P_{spank}-*kinA lacI* $spec^R$] *sacA*::[P_{ald}-*ald-mcherry lacI* kan^R]	BIB1332 transformed with EIB503
**BIB1442**	168 $P_{hyperspank}$-*rapA* P_{ald}-*ald-mcherry*	*trpC2* *amyE*::[$P_{hyperspank}$-*rapA lacI* $spec^R$] *sacA*::[P_{ald}-*ald-mcherry lacI* kan^R]	BIB1330 transformed with EIB503

Supplementary Table 4. Primers used in this work

Primer	Sequence 5' – 3' (LIC-site underlined; overlap in gray italics; Restriction cut-site in bold)	Purpose
**ST162**	CATAGTAGTTCCTCCTTCCCGGGAAAGC	pRFP_Star
**ST219**	*CGGGAAGGAGGAACTACT*ATGGTTTCCAAGGGCGAGG	pRFP_Star
**ST220**	*CCAAGCTCAGCTAATTAAGC*TTATTTGTACAGCTCATCCATGCCAC	pRFP_Star
**ST221**	GCTTAATTAGCTGAGCTTGGACTCC	pRFP_Star
**ST1**	<u>CCGCGGGCTTTCCCAGC</u>TAAAAGAAAGCACGGGTGTTTG	PrapAII up fragment
**ST222**	*AATCGTCTGCTTCATCCT*TTTGAATTACCCGAGATATGTC	PrapAII up fragment
**ST223**	AGGATGAAGCAGACGATTCCGTC	PrapAII down fragment
**ST224**	<u>GTTCCTCCTTCCCACC</u>CCTTCGATGTCTTCTAACAATTCTG	PrapAII down fragment
**ST209**	<u>CCGCGGGCTTTCCCAGC</u>TCCTAACAAATCGGTTTCTC	PspoIIE
**ST210**	<u>GTTCCTCCTTCCCACC</u>ACCTGTTATATTCGTTGCCTG	PspoIIE
**ST16**	GGTAAGTTTTCCGTATGTTGC	Seq pGFP_Star
**ST17**	GTGAATTTAGGAGGCTTACTTG	Seq pXFP_Star
**ST243**	GTCTGGGTGCCTTCATAC	Seq pRFP_Star
**ST133**	TCAGCTCGCATACCGAATTAAG	PtrpE-mcherry
**ST134**	ATGCCTAAACACCAGCCATCC	PtrpE-mcherry
**SS34**	**TATATAGCTAGC**ATATGACATATCTCGGGTAATTCAAAAGG	rapA_fw
**SS37**	**TATATAGCATGC**GACCGCAACGAGCAACAAACCTGACATC	rapA_rev
**MA34**	<u>CCGCGGGCTTTCCCAGC</u>GCGGGCCATTATAATTACTC	Pald_fw
**MA35**	<u>GTTCCTCCTTCCCACC</u>CCTGTATATGTGATATTTTTAGTGTAGC	Pald_rev
**MA37**	**ATAGCTAGC**TATCACATATACAGGAGGAGACAG	ald_fw
**MA38**	**ATAGCATGC**GTCATAATTCGTGAAATGGTCTC	ald_rev
**MA47**	**ATAACTAGT**TGGCCTTTTTGCGTTTCTAC	Phyperspank_ald_fw
**MA48**	**ATAGAGCTC**TTTCCTTACGCGAAATACGG	Phyperspank_ald_rev
**SONSEQ18**	ATGGCAAGAACGTTGCTCGA	Seq pDR111
**SONSEQ19**	TACGTACGATCTTTCAGCCG	Seq pDR111
**MA49**	GTGACGATCATTGACTTAAACG	Seq Phyp_ald

**MA50**	ATATTCAAACGGAGGGAGACG	Seq Phyp_ald
**ST39**	CTGGTCGGAGATTGGGATGATAG	Check for chromosomal integration into *amyE* locus
**ST40**	AATTTCCATGTTGCGTAAGTCAG	Check for chromosomal integration into *amyE* locus
**ST283**	GCTCAATATGTCGTCATTACAGGC	Check for chromosomal integration into *sacA* locus
**ST284**	GCAGGGCTAATTGCAGATATAGG	Check for chromosomal integration into *sacA* locus
**ST129**	ATGAGTATTCAACATTTCCGTGTC	Check for single crossover
**ST130**	TTACCAATGCTTAATCAGTGAGG	Check for single crossover
**MA51**	GATGAAGATCTGCTGACATTG	ald up_f
**MA52**	CGAGCGCCTACGAGGAATTTGTATCGGGAACCC CTATGATCATATC	ald up_r
**MA53**	CCTATCACCTCAAATGGTTCGCTGGATCTAGGCT ATGAGTATGTTCC	ald down_f
**MA54**	CCAAACAGCCTAAAGACTG	ald down_r
**LA54**	CAGCGAACCATTTGAGGTGATAGGTCTTGCAAT GGTGCAGGTTGTTCTC	Tet fwd
**LA56**	CGATACAAATTCCTCGTAGGCGCTCGGGAACTC TCTCCCAAAGTTGATCCC	Tet rev
**LA58**	CATCGGTCATAAAATCCGTAATGC	Tet check rev
**KN1**	TCATATCAGCGACGTTCTGC	Ald-up-check
**KN4**	CTGGTGCTGGAATGAGTTTGCT	Tet check fwd
**KN3**	AAATGCGGATGGCACATATT	Ald-do-check
**MA55**	**ATAGGTACC**GCGGGCCATTATAATTACTC	Pald_ald fw
**MA56**	*GGAACCTCCACCTCC*AGCACCCGCCACAGATGAT	Pald_ald rev
**MA57**	*GTGCTGGAGGTGGAGGTTCC*GTTTCCAAGGGCGA GGAG	mcherry fw
**MA58**	**ATAGGATCC**TTATTTGTACAGCTCATCCATGCC	mcherry rev

Supplementary References

1. Trauth, S. & Bischofs, I. B. Ectopic integration vectors for generating fluorescent promoter fusions in *Bacillus subtilis* with minimal dark noise. *PLoS One* **9,** e98360 (2014).

2. Middleton, R. & Hofmeister, A. New shuttle vectors for ectopic insertion of genes into *Bacillus subtilis*. *Plasmid* **51,** 238–245 (2004).

3. Guérout-Fleury, A. M., Shazand, K., Frandsen, N. & Stragier, P. Antibiotic-resistance cassettes for *Bacillus subtilis*. *Gene* **167,** 335–336 (1995).

4. de Jong, I. G., Veening, J.-W. & Kuipers, O. P. Heterochronic phosphorelay gene expression as a source of heterogeneity in *Bacillus subtilis* spore formation. *J. Bacteriol.* **192,** 2053–67 (2010).

5. Levine, J. H., Fontes, M. E., Dworkin, J. & Elowitz, M. B. Pulsed feedback defers cellular differentiation. *PLoS Biol.* **10,** e1001252 (2012).
