# Supplementary material for: Phenotypic memory in Bacillus subtilis links dormancy entry and exit by a spore quantity-quality tradeoff
Source: Nat Commun. 2018 Jan 4;9:69. doi: 10.1038/s41467-017-02477-1 (PMC5754360; doi:10.1038/s41467-017-02477-1)
Supplement: Supplementary file 3 — Description of Additional Supplementary Files [file 41467_2017_2477_MOESM3_ESM.pdf]

Description of Additional Supplementary Files

File Name: Supplementary Movie 1
Description: **Heterochronic sporulation response of B. subtilis on an SM* gel pad and the subsequent spore revival upon addition of L-alanine.** The strain BIB1019 carries PtrpE*-mCherry and PspoIIE-gfp reporters. After 90 h of starvation, spores have been released from the mother cell. To visualize the distribution of early and late spores prior to the nutrient up-shift, false colored frames were included with early and late spores denoted in yellow and green, respectively. Note that most spores geminated, but only early spores succeed in resuming vegetative growth. Spores that grew out upon nutrient stimulation are circled in red. http://doi.org/10.5446/33993

File Name: Supplementary Movie 2
Description: **The down- and up-shift responses on an SM* gel pad of the fluorescent marker strain BIB1126, which carries a PrapA-mCherry promoter fusion that reports on sporulation timing.** A few cells that initiated sporulation early were tracked with a yellow arrow, while some representative cells that delayed sporulation were followed with a green arrow. The latter strongly induce mCherry expression. After completion of sporulation, the marker nicely distinguishes between early and late spores. Spore revival was then induced by the addition of L-alanine. All spores that grew out in response to induction with L-alanine are circled. They all showed low fluorescence. http://doi.org/10.5446/33994

File Name: Supplementary Movie 3
Description: **Up-shift response of spores of the fluorescent marker strain PrapA-mCherry upon exposure to L-alanine**. Spores of BIB1126 were generated by the Sterlini-Mandelstam protocol in liquid shake-flask culture, and showed a broadly heterogeneous distribution of fluorescence. Spores were then spotted onto an SM gel pad and spore revival was induced by adding Lalanine. http://doi.org/10.5446/33995

File Name: Supplementary Movie 4
Description: **Effect of an ald gene knock-out**. Down- and up-shift responses of a mutant colony of strain BIB1416 (Δald, PtrpE-mCherry) and a non-fluorescent WT colony BIB224 was monitored. Both strains were starved in co-culture on an SM* gel pad and showed comparable sporulation dynamics. After sporulation was complete, spore revival was induced with L-alanine. Both mutant and WT spores showed comparable germination. However, only WT spores grew out, while the mutant spores did not. https://doi.org/10.5446/34001

File Name: Supplementary Movie 5
Description: **Effect of Ald re-programming on B. subtilis spore revival**. Down- and up-shift responses of strain BIB1300, which harbors a PrapA-mCherry promoter fusion that reports on sporulation timing and in which Ald expression can be controlled by an IPTG-inducible promoter was monitored. Cells were spotted onto an SM* gel pad. At the indicated time IPTG was added to the pad. This resulted in the expression of Ald in the progenitor cells of late spores. After sporulation was complete, spore revival was induced with L-alanine. The spores that will grow out are circled. Note that red-fluorescent late spores were able to grow out successfully. http://doi.org/10.5446/33996

File Name: Supplementary Movie 6
Description: **C-terminal mCherry-tagged Ald expression from its native promoter**. The down- and upshift responses of strain BIB1423, which expresses fluorescently tagged Ald-mCherry from its native promoter (amyE::Pald-ald-mCherry) was monitored. At the onset of sporulation, cells that

sporulated early had higher fluorescence levels than cells that delayed sporulation due to dilution. The fluorescence was carried from the progenitors into the developing spore. Upon spore release, fluorescence dropped due to unknown reasons and then remained stable. Early spores showed higher fluorescence than late spores. All spores that grew out in response to Lalanine stimulation were circled. They all showed higher levels of fluorescence compared to nonoutgrowing spores. Note that BIB1423 also carries Pald-gfp. The resulting fluorescence from the green fluorescence channel is not shown for clarity. http://doi.org/10.5446/33997

File Name: Supplementary Movie 7
Description: **Effect of accelerating sporulation via overexpression of KinA**. Down- and up-shift responses of a mutant colony of strain BIB1332 (Pspank-kinA) and a WT colony BIB1126 (PrapAmCherry) was monitored. Both strains were starved in co-culture on a SM* gel pad, which was supplemented with IPTG to induce KinA in the mutant strain and accelerate its sporulation. After sporulation was complete, spore revival was induced with L-alanine. http://doi.org/10.5446/33998

File Name: Supplementary Movie 8
Description: **Effect of decelerating sporulation via overexpression of RapA.** Down- and up-shift responses of a mutant colony of strain BIB1330 (Phyperspank-rapA) and a WT colony BIB1126 (PrapA-mCherry) was monitored. Both strains were starved in co-culture on a SM* gel pad that was supplemented with IPTG to induce RapA in the mutant strain and decelerate its sporulation. http://doi.org/10.5446/33999

File Name: Supplementary Movie 9
Description: **Effect of KinA induction on sporulation and spore revival of the Pald Ald-mCherry reporter strain**. Left: KinA was induced at t = 0 resulting in accelerated sporulation of BIB1440. All KinAinduced spores are highly fluorescent and most were capable of outgrowth in response to Lalanine. Right: The induction of KinA was delayed (t = 20h) and induced in the progenitor cells of late spores. While the highly fluorescent early spores grew out, none of the resulting lowly fluorescent, late but KinA-induced spores grew out. This shows that KinA induction does not alter the spore properties per se. http://doi.org/10.5446/34000
